# The Effects of Thiamine on Breast Cancer Cells

**DOI:** 10.3390/molecules23061464

**Published:** 2018-06-16

**Authors:** Xiaowen Liu, Sophia Montissol, Amy Uber, Sarah Ganley, Anne V. Grossestreuer, Katherine Berg, Stanley Heydrick, Michael W. Donnino

**Affiliations:** 1 Department of Emergency Medicine, Beth Israel Deaconess Medical Center, Harvard Medical School, Boston, MA 02115, USA; xliu4@bidmc.harvard.edu (X.L.); smontiss@bidmc.harvard.edu (S.M.); aj.uber@gmail.com (A.U.); sganley26@gmail.com (S.G.); agrosses@bidmc.harvard.edu (A.V.G.); kberg@bidmc.harvard.edu (K.B.); sheydric@bidmc.harvard.edu (S.H.); 2 Department of Medicine, Division of Pulmonary and Critical Care, Beth Israel Deaconess Medical Center, Harvard Medical School, Boston, MA 02115, USA

**Keywords:** thiamine, breast cancer, aerobic glycolysis, aerobic metabolism, proliferation, apoptosis

## Abstract

(1) Background: Thiamine is an important cofactor for multiple metabolic processes. Its role in cancer has been debated for years. Our aim is to determine if thiamine can convert the cellular metabolic state of breast cancer cells from anaerobic to aerobic, thus reducing their growth. (2) Methods: Breast cancer (MCF7) and non-tumorigenic (MCF10A) cell lines were treated with various doses of thiamine and assessed for changes in cell growth. The mechanism of this relationship was identified through the measurement of enzymatic activity and metabolic changes. (3) Results: A high dose of thiamine reduced cell proliferation in MCF7 (63% decrease, *p* < 0.0001), but didn’t affect apoptosis and the cell-cycle profile. Thiamine had a number of effects in MCF7; it (1) reduced extracellular lactate levels in growth media, (2) increased cellular pyruvate dehydrogenase (PDH) activities and the baseline and maximum cellular oxygen consumption rates, and (3) decreased non-glycolytic acidification, glycolysis, and glycolytic capacity. MCF10A cells preferred mitochondrial respiration instead of glycolysis. In contrast, MCF7 cells were more resistant to mitochondrial respiration, which may explain the inhibitory effect of thiamine on their proliferation. (4) Conclusions: The treatment of MCF7 breast cancer cells with 1 μg/mL and 2 μg/mL of thiamine for 24 h significantly reduced their proliferation. This reduction is associated with a reduction in glycolysis and activation of the PDH complex in breast cancer cells.

## 1. Introduction

First described by Otto Warburg in 1956, it has long been known that that cancer cells favor anaerobic over aerobic metabolism to produce energy to fuel tumor growth. Now termed the Warburg effect, this effect appears to hold true whether or not oxygen is present, and can thus be termed aerobic glycolysis [1]. Overall, this metabolic shift causes cancer cells to have a higher rate of glucose metabolism, greater lactate production, and an enhanced biosynthesis of lipids and other macromolecules [1,2].

Thiamine (vitamin B1), an essential cofactor for several key enzymes, is responsible for metabolic processes including biomass generation, amino acid catabolism, and the generation of energy [3]. Most notably, thiamine acts as an effective gatekeeper of aerobic metabolism in its role as cofactor for pyruvate dehydrogenase, the enzyme that allows pyruvate to enter the Krebs cycle. The role of thiamine in cancer has been debated for years. Tsao et al. recognized a substantial reduction of thiamine pyrophosphate in whole blood from patients diagnosed with advanced stages of non-small cell lung cancer [4]. A 2001 study by Comin-Anduix et al. showed a biphasic effect of thiamine when administered to mice injected with Ehrlich’s ascites tumor cells. They reported increased tumor proliferation when mice were given lower doses of thiamine and decreased tumor proliferation when mice were given 2500 times the recommended daily allowance (RDA) of thiamine for mice for eight days [5]. Interestingly, the proliferative effect of low doses of thiamine was diminished, and the anti-proliferative effect of high doses of thiamine was increased if the mice were pretreated with either low or high does thiamine for seven days prior to the introduction of tumor cells. However, this same study, as well as others, found inconsistent results when trying to associate cancer with dietary intake of thiamine [5,6,7,8,9]. For example, a 2008 study following a large cohort of women over 16 years found no link between the intake of B vitamins and cancer [7]. In contrast, other animal and human studies imply that thiamine deficiency is related to tumor growth [8,9].

When pyruvate dehydrogenase (PDH) activity is depressed due to thiamine deficiency or high phosphorylation by the overexpression of pyruvate dehydrogenase kinase (PDK) [10,11], aerobic glycolysis will be employed in the place of failed aerobic metabolism. When thiamine is administered to severely deficient patients, aerobic metabolism is rapidly restored, and the clearance of lactate and reversal of shock can occur within hours. Since tumor cells prefer aerobic glycolysis for tumor growth, the restoration of aerobic metabolism could theoretically slow the growth of the tumor. This hypothesis has been tested previously with dichloroacetate, which is another regulator of PDH. Dichloroacetate was found to attenuate glycolysis and increase aerobic metabolism, resulting in the reduction of colorectal cancer cell growth [12]. However, dichloroacetate also has some potentially toxic effects, including significant neuropathy, which may make it unsuitable for use in cancer patients.

In this study, we hypothesized that the administration of thiamine to breast cancer cells would convert their metabolic phenotype from aerobic glycolysis to aerobic metabolism, and thus reduce cellular proliferation. Thiamine has a distinct advantage over drugs such as dichloroacetate because it is a water-soluble vitamin that is harmless, even in excess. Moreover, we have already shown that administration of thiamine to thiamine-deficient patients can quickly cause a whole-body shift from aerobic glycolysis toward aerobic metabolism [13]. To test our hypothesis, we conducted an experiment in which we incubated breast cancer cell line MCF7 and non-tumorigenic MCF10A human mammary breast epithelial cells with varying concentrations of thiamine, and assessed their rates of growth and metabolic phenotypes.

## 2. Results

### 2.1. High Doses of Thiamine Reduced Cell Vialbility Over Time in Breast Cancer Cells, But Not in Normal Breast Epithelial Cells

**Figure 1:**
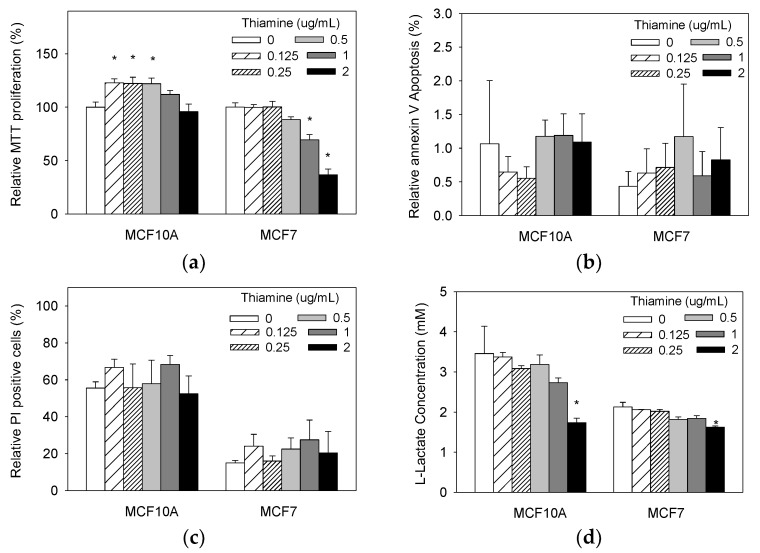
(**a**) Thiamine (1 μg/mL and 2 μg/mL) did not significantly reduce growth of cultures of non-tumorigenic MCF10A cells, but did cause a significant reduction in the growth of cultures of breast cancer MCF7 cells (*p* < 0.05). (**b**) % of cells that were Annexin-V positive. (**c**) % of cells that were propidium iodide (PI) staining positive. (**d**) Thiamine reduced lactate levels in growth media in a dose-dependent manner in both cancer and non-tumorigenic cells. Cells were treated with various doses of thiamine or vehicle control, and the relative number of viable cells was assessed at 24 h using MTT assay for (**a**) Annexin-V assay for (**b**) and propidium iodide assay for (**c**). Data are expressed as percentage of control (0 μg/mL thiamine) for (**a**–**c**). Extracellular lactate levels were measured in the growth media using a L-lactate assay kit for (**d**). Results are expressed as means ± SE (* significant difference relative to control (0 μg/mL thiamine supplementation), white bar).

First, we determined whether supplementing media containing a baseline level of thiamine with increasing doses of thiamine hydrochloridedifferentially affected cellular proliferation in cancer and non-tumorigenic wild-type epithelial cells. After 24 h of thiamine treatment, we found that there was no significant difference in the progression of non-tumorigenic MCF10A cultures, using 1 μg/mL and 2 μg/mL of thiamine, while thiamine additions of 0.125 μg/mL, 0.25 μg/mL, and 0.5 μg/mL significantly increased cell viability over time for non-tumorigenic MCF10A cells (Figure 1a). Conversely, treatment using increasing doses of thiamine reduced cell viability overtime in a dose-dependent manner in breast cancer MCF7 cells (Figure 1a). No significant effect of 0.125 μg/mL, 0.25 μg/mL, and 0.5 μg/mL thiamine was found on thethe proliferation of cancerous MCF7. However, the administration of 1 μg/mL and 2 μg/mL thiamine significantly reduced the growth of breast cancer MCF7 cultures (*p* = 0.04, *p* < 0.0001, respectively). The growth of MCF7 cells treated with 2 μg/mL thiamine decreased up to 63% compared to cells treated with vehicle control.

### 2.2. Thiamine Did Not Affect Apoptosis in Both Breast Cancer Cells and Non-Tumorigenic Cells

Next, we investigated whether the reduced growth of cultures with thiamine treatment was associated with an induction of apoptosis. Cells were treated with increasing doses of thiamine hydrochloride (0 μg/mL, 0.25 μg/mL, 0.5 μg/mL, 1 μg/mL, and 2 μg/mL) for 24 h, and the proportion of cells undergoing apoptosis was assessed by detecting membrane phosphatidylserine with Annexin V-FITC. Cells were stained with Annexin V-FITC and vital dye 7-AAD, and analyzed using flow cytometry. No significant induction of apoptosis in the cancer cell lines after 24 h of treatment in any dose was found (Figure 1b). Similar results were found in the non-tumorigenic cells.

We also examined whether the reduction in growth of cultures with thiamine treatment was associated with an induction of growth arrest and subsequent necrosis. Cells were treated with 2 μg/mL thiamine for 24 h, and cell-cycle profiles were analyzed using a flow cytometric assessment of DNA content after propidium iodide (PI) staining. Thiamine treatment did not cause significant changes in PI incorporation into either MCF7 cancer cells or the non-tumorigenic MCF10A cells (Figure 1c).

### 2.3. Thiamine Reduced Extracellular Lactate Levels in Growth Media of Both Breast Cancer Cells and Non-Tumorigenic Cells

We subsequently measured growth media lactate levels at the end of the experiment (24 h) to test whether the changes in growth induced by thiamine is correlated with reduced glycolysis. Lactic acid is the end product of glycolysis. If thiamine induced mitochondrial oxidative phosphorylation, pyruvate would be decarboxylated to acetyl coenzyme A and not be reduced to lactate, leading to a decrease in lactate levels in the growth media. Lactate levels in the growth media of all of the cell lines were measured after 24 h of treatment with increasing doses of thiamine. A downward trend in endpoint media lactate levels was observed with increasing doses of thiamine for both MCF7 cancer cells and non-tumorigenic MCF10A cells. However, this trend was more pronounced with MCF7 cells, especially at the highest thiamine concentration (Figure 1d).

### 2.4. Thiamine Increased Cellular PDH Activities in Breast Cancer Cells

**Figure 2:**
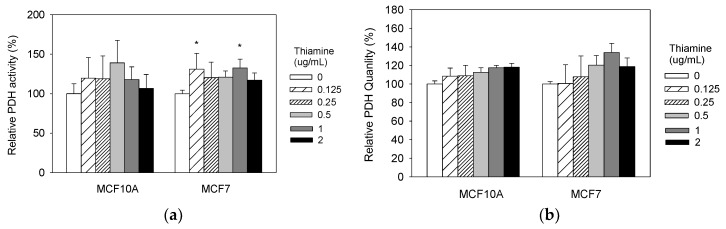
Thiamine increases cellular pyruvate dehydrogenase (PDH) activities in breast cancer cells. Cells were treated with range of doses of thiamine for 24 h, and PDH activity (**a**) and quantity (**b**) were then measured using the PDH combo microplate assay kit (Abcam, Inc., Cambridge, MA, USA). Results are expressed as mean ± SE (* significant difference relative to control (0 μg/mL Thiamine supplementation), white bar).

To test whether the changes in growth induced by thiamine are due to activation of the PDH complex, we measured PDH activity and quantity after treating both cell lines with increasing doses of thiamine for 24 h. PDH complexes were solubilized from mitochondria, and then immune-captured in 96 well plates. The activity and quantity were determined. Treatment with 0.125 μg/mL and 1 μg/mL thiamine significantly increased PDH activity levels in breast cancer MCF7 cell line (Figure 2), but not in non-tumorigenic MCF10A cells.

### 2.5. High Doses of Thiamine Increased Baseline and Maximum Cellular Oxygen Consumptions in Breast Cancer Cells

**Figure 3:**
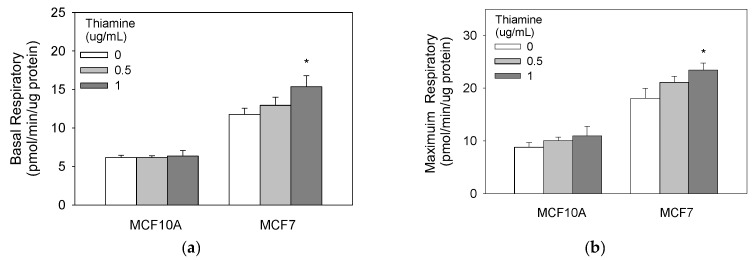
Thiamine increases baseline (**a**) and maximum (**b**) cellular oxygen consumption in breast cancer cells. Cells were treated with a range of thiamine doses for 24 h before subjecting them to a mitochondria stress test (Seahorse Bioscience, Billerica, MA). Results are expressed as mean ± SE (* significant difference relative to control—white bar (0 μg/mL thiamine).

We next investigated the metabolic changes induced by thiamine in both breast cancer and non-tumorigenic cells using Seahorse XFe96 oxygen consumption analyzer. These mitochondrial stress tests indicate that 1 μg/mL of thiamine supplementation over 24 h significantly increased basal, maximum, and ATP production oxygen consumption rate for the MCF7 but not in non-tumorigenic MCF10A cells (Figure 3).

### 2.6. High Doses of Thiamine Decreased Non-Glycolytic Acidification, Glycolysis, and Glycolytic Capacity in Breast Cancer Cells

**Figure 4:**
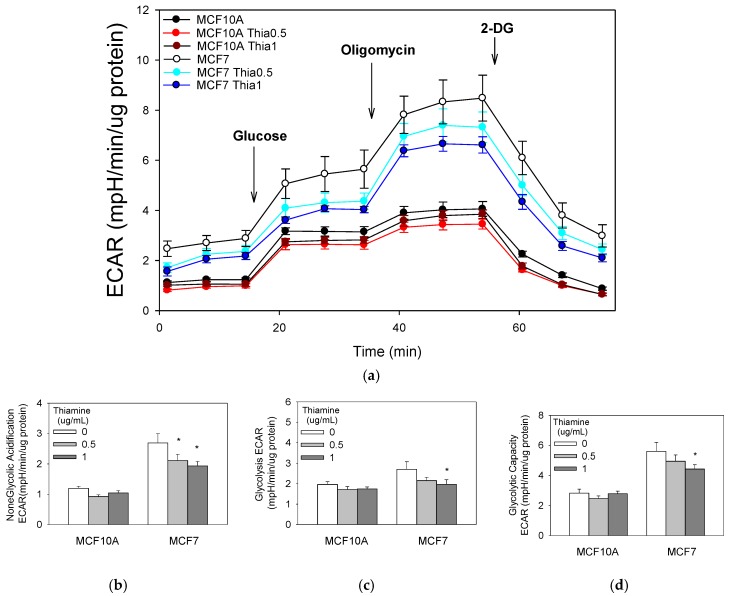
Glycolysis stress test **(a)**. Cells were treated with a range of doses of thiamine for 24 h before a glycolysis stress test (Seahorse Bioscience, Billerica, MA, USA). Thiamine decreases non-glycolytic acidification (**b**), glycolysis (**c**), and glycolytic capacity (**d**) in breast cancer cells. Results are expressed as mean ± SE (* significant difference relative to control (0 μg/mL Thiamine supplementation), white bar).

We also investigated the extracellular acidification rate (ECAR) coupled to glycolysis. Both cell types were starved of glucose for two hours; then, the change in medium acidification was measured after administration of a bolus of glucose. Breast cancer MCF7 cells ECAR after 2 h of glucose starvation were approximately twofold greater than the non-tumorigenic MCF10A cells. Immediately following the addition of glucose, glycolytic ECAR was measured. Extracellular acidification in MCF7 cells occurred at a significantly greater rate compared with non-tumorigenic MCF10A cells (Figure 4). Cellular glycolytic capacity was determined by adding oligomycin, an inhibitor of ATP synthase, to the cell cultures. After this treatment, cells reduced their mitochondrial respiration and maximized their glycolic ATP production. Glycolytic capacity and reserve glycolytic capacity significantly increased in MCF7 cell cultures compared with MCF10A cells, further suggesting that breast cancer cells modify themselves to satisfy ATP energetic demands by acquiring a glycolytic phenotype. The addition of 2-deoxyglucose (2-DG), an inhibitor of the first step of glycolysis, confirmed that the ECAR measured was a result of glycolytic metabolism. Following 2-DG treatment, ECAR was restored to non-glycolytic levels in both cell lines. More importantly, 0.5 μg/mL and 1 μg/mL of thiamine treatment for 24 h significantly decreased the non-glycolytic acidification in breast MCF7 cancer cells, but not in non-tumorigenic cells (Figure 4). 

### 2.7. High Dose of Thiamine Altered Cell Energy Phenotypes of Breast Cancer and Non-Tumorigenic Cells

To better understand which energy pathways the breast cancer cell line MCF7 and the non-tumorigenic MCF10A cells prefer, we generated a functional overview of their metabolic phenotypes. All living cells have an energy phenotype, which compares the relative utilization of both mitochondrial respiration and glycolysis to meet cellular energy requirements. An energy phenotype comprises a baseline phenotype, a stressed phenotype, and a metabolic potential. The metabolic potential reveals the cells’ ability, and preferred pathway, to respond to stress-induced changes in energy demand. The metabolic potential is quantified as the difference between the baseline phenotype and the stressed phenotype.

**Figure 5:**
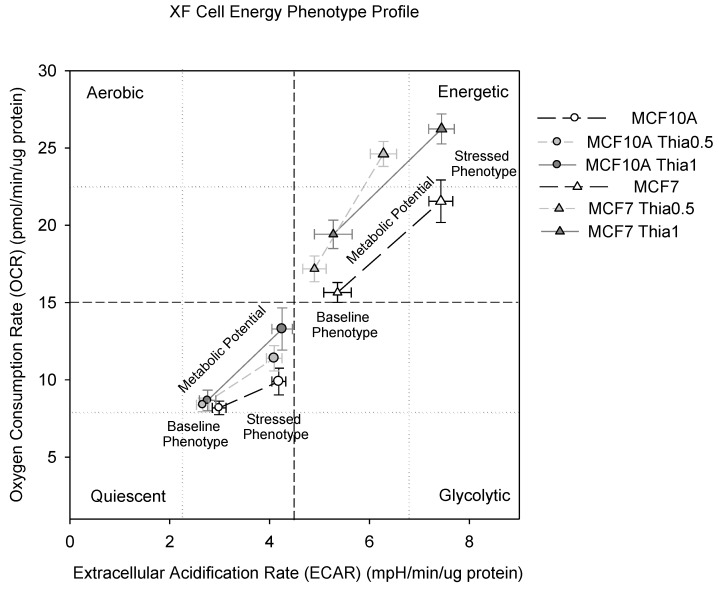
Cell Energy Phenotyping. Cells were treated with a range of doses of thiamine for 24 h before cell energy phenotyping (Seahorse Bioscience, Billerica, MA).

Non-tumorigenic MCF10A cells were more quiescent at baseline and had less metabolic potential compared with breast cancer MCF7 cells. After 24 h of thiamine treatment, the baseline phenotype revealed that both of the cell lines increased mitochondrial respiration and maintained/decreased glycolysis. During stress, 24 h of thiamine treatment increased the metabolic potential of non-tumorigenic MCF10A cells, but not of MCF7 cells (Figure 5). The non-tumorigenic MCF10A cells preferred the mitochondria pathway instead of the glycolysis. The MCF7 cells are more resistant to mitochondrial respiration, which may explain the inhibition of the proliferation of cancer cells.

## 3. Discussion

In this study, we found that treatment of breast cancer MCF7 cells with high doses of thiamine over 24 h resulted in a significant reduction in the number of viable cells compared to untreated controls, but thiamine treatment did not affect the number of viable non-tumorigenic MCF10A cells. Since there was no significant effect of thiamine on the induction of apoptosis/necrosis or growth arrest on either cell-type, we conclude that thiamine’s effect on MCF7 cells was likely due to an inhibitory effect on cell proliferation. Breast cancer cells were highly glycolytic compared to non-tumorigenic cells. When treated with thiamine, they showed decreased lactate levels in the growth media, suggesting that glycolysis was reduced. Moreover, PDH activity was increased in breast cancer cells, which is consistent with thiamine’s action on this enzyme, and likely serving as the mechanism for the observed metabolic and proliferative changes in the cells. Thiamine treatment also led to less glycolytic and more aerobic cells on energy phenotype mapping in cancerous cells. In contrastthiamine had no significant effect on cell proliferation or PDH activity in MCF10A cells.

Tumor cells shift between glycolysis and mitochondrial metabolism to adapt to different microenvironments [14,15,16] in order to meet cellular energy requirements for increased cancer growth and proliferation [17]. Targeting mitochondrial bioenergetics and the glycolytic pathway has been an area of interest for chemotherapeutic strategy in recent years [17]. To fully contextualize the biochemistry of metabolism as it relates to tumor cell growth and proliferation, it helps to first consider the metabolism of normal, quiescent cells, such as non-tumorigenic MCF10A cells. These cells have a relatively low basal rate of glycolysis; the glycolysis-derived pyruvate predominantly enters the mitochondrial matrix, where it is oxidized to acetyl CoA by PDH complex. Acetyl CoA then feeds into the TCA cycle, followed by oxidative phosphorylation for high-efficiency ATP generation. However, in cancer cells such as breast cancer MCF7, rapid cell proliferation requires an accelerated production of basic cellular building blocks in order to assemble new cells; cellular metabolism alterations feed tumor growth by maximally generating substrates for biosynthesis. Increases in glucose consumption produce increases in intermediate glycolytic metabolites as precursors for nucleotide, amino acid, and lipid synthesis [18], and a significant amount of ATP from glycolysis. Following glycolysis, most pyruvate is converted to lactate in the cytoplasm and secreted, instead of being oxidized through mitochondrial metabolism.

While the literature on this topic has shown mixed results, we believe that our findings and those of others may be reconciled by considering the level of thiamine in cancerous cells and the dose of thiamine used in prior experiments. Trebukhina et al. found that tumor growth resulted in tissue vitamin store depletion [19], and that during tumor growth, cancer cells kept a constant level of TPP, while host liver tissue exhibited a continuous decline [20]. Another study, analyzing post-surgical or autopsy tissues, found that thiamine levels increased 2.5-fold in colon adenocarcinomas relative to uninvaded control tissue [21]. These studies strongly suggest preferential thiamine accrual in cancer cells. This accumulation of thiamine may account for the effectiveness of a low dose of thiamine supplementation in promoting cancer growth, which has been shown in an animal study [5]. We hypothesize that a low dose may only be enough to provide only the basic cellular building blocks for assembling new cells; however, a high dose of exogenous thiamine may provide not just the cellular building blocks, but may preferentially shift metabolism from glycolytic to aerobic via reactant loading. By promoting aerobic mitochondrial metabolism, fewer glycolytic intermediates and micronutrient building blocks are generated, and cancer cell proliferation is thus stunted. This theory is supported by Hanberry et al., who found that a high dose of thiamine reduces cancer cell proliferation at a level similar to dichloroacetate by reducing PDH phosphorylation, glucose consumption, lactate production, and mitochondrial membrane potential [22]. Our findings of slightly increased PDH activity, decreased lactate, and more aerobic energy phenotype mapping in cancer cells treated with thiamine further support this hypothesis.

An important caveat to our conclusions is the assumption that the phenotypes of the MCF7 and MCF10A cells lines that we used mimic the phenotype of breast ductal cell cancer cells and normal breast ductal cells, respectively. MCF7 cells were derived from a human breast tumor. Similar to their expected behavior in tumors, they grew rapidly and were not contact inhibited. MCF10A cells were isolated from human breast duct cells, but were then transformed to allow long-term passaging. However, they appeared to retain at least some of the characteristics of wild-type ductal cells, since they grew only to confluent monolayers.

The results shown in this study are important in defining and advancing the role of thiamine in cancer by showing a significant reduction in the growth of breast cancer cells when treated with thiamine. In the clinical setting, slowing the growth of cancer cells may provide more time to explore treatment options, increase the array of surgical options, and help prevent the spread of cancer. In the laboratory setting, the ability to reduce cancer growth is a significant step in the exploration for a cure for cancer. Further studies are warranted to establish dosing guidelines under which thiamine reduces tumor cell growth in vivo, and investigate the therapeutic benefits of combining appropriate levels of thiamine with other pharmaceutical components of the standard care.

## 4. Materials and Methods

### 4.1. Cell Cultures

MCF10A (human mammary gland/breast epithelial cells from non-cancer origin) and MCF7 (human mammary gland epithelial cells from metastatic site) were acquired from the American Type Culture Collection (ATCC, Manassas, VA, USA). MCF10A cells were kept in base medium mammary epithelial cell basal medium (MEBM) along with the additives kit (MEGM kit) purchased from Lonza (Portsmouth, NH, USA) and 100 ng/mL cholera toxin. The GA-1000 (gentamycin-amphotericin B mix) was not used from the MEGM kit, as suggested by ATCC’s protocol. MCF7 cells were maintained in Eagle’s Minimal Essential Medium (EMEM) medium, which was supplemented with 10% fetal calf serum (FBS). Both cells were kept in a 37 °C, 5% CO_2_ humidified incubator and subcultured according to the manufacturer’s instructions. The “baseline” thiamine concentration in MEBM is 0.337 μg/mL (1 µM) (Lonza technical support), and that in un-supplemented EMEM is 1.0 μg/mL (3 µM). FBS has an average thiamine concentration of 0.1 μg/mL. (HyClone technical support), so 10% FBS supplementation gave a final thiamine concentration of ~0.91 μg/mL (2.7 µM) in the MCF7 growth medium ((10% × 0.1) + (90% × 1.0)). Thiamine hydrochloride was purchased from Sigma (Saint Louis, CA, USA).

### 4.2. MTT Cell Proliferation Assays

Vybrant^®^ MTT Cell Proliferation Assay Kits were purchased from Life technologies (Carlsbad, CA, USA). 1 × 10^4^ cells were seeded in a 96-well tissue culture plate per well. After incubating the plate overnight, fresh media containing additional increasing doses of thiamine hydrochloride (0 μg/mL, 0.125 μg/mL, 0.25 μg/mL, 0.5 μg/mL, 1 μg/mL, and 2 μg/mL) were added to each well. After 24 h of incubation, the medium in the 96-well plates was replaced with 50 mL of 1 mg/ mL of MTT (3-(4,5-dimethylthiazol-2-yl)-2,5-diphenyltetrazolium bromide) solution. The plates were then incubated in the dark for 3 h. The MTT solution was removed, and the purple formazan precipitates were dissolved in 100 mL of propanol. Optical density was measured using a microplate reader SpectraMAX190 (molecular devices, Sunnyvale, CA, USA) at 570 nm.

### 4.3. Annexin V and Propidium Iodide Apoptosis Assays

An Annexin-V apoptosis detection kit and propidium iodide staining solution were purchased from eBioscience (San Diego, CA, USA). Cells were seeded in six-well tissue culture plates in standard conditions. After overnight incubation, fresh media containing additional increasing doses of thiamine hydrochloride (0 μg/mL, 0.125 μg/mL, 0.25 μg/mL, 0.5 μg/mL, 1 μg/mL, and 2 μg/mL) were added to each well. Flow cytometric analysis was performed after 24 h of incubation. The cells were washed twice with cold phosphate buffered saline (PBS), and resuspended in binding buffer at 5 × 10^6^ cells per mL. One hundred μL of cell suspension (5 × 10^5^ cells) was transferred to 5-mL culture tubes. These cells were stained with 5 μL of Annexin V-FITC, gently vortexed, and incubated at ambient temperature for 15 min in the dark. Next, cells were washed and resuspended in 200 μL of binding buffer. Then, 5 μL of propidium iodide staining solution was then added to the tube. Stained cells were analyzed within an hour on an LSR II flow cytometer (BD Bioscience, San Jose, CA, USA). FlowJo software (FlowJo, Ashland, OR, USA) was used to analyze the data.

### 4.4. Lactate Levels

Lactate is generated as a byproduct of aerobic glycolysis, and was measured to provide insight on the type of energy generation in cell cultures. Lactate measurements were performed in growth medium using the l-lactate assay kit from Eton Bioscience (San Diego, CA, USA). Media collected from above assays were stored in −80 °C Lactate levels were measured according to the manufacturer’s instructions. The sensitivity of the assay is 60 μM to 3000 μM.

### 4.5. PDH Activity and Quantity Assay

PDH is the gatekeeper enzyme for the Krebs cycle, and is therefore a good proxy for aerobic metabolism. To determine PDH activity and quantity, cells were treated with additional doses of thiamine as described above. After 24 h of treatment, the cells were harvested in PBS supplemented with proteinase inhibitor. After collection, cell pellets were immediately frozen in liquid nitrogen, and stored at −80 °C until assayed. On the day of the assay, cells were resuspended in ice-cold PBS supplemented with a proteinase inhibitor. PDH activity and quantity were then assessed using the PDH combo microplate assay kit (Abcam, Inc., Cambridge, MA, USA) in accordance with the manufacturer’s protocol.

### 4.6. Oxygen Consumption and Extracellular Acidification Measurements

Real-time oxygen consumption rate (OCR) and extracellular acidification rates (ECAR) were measured using the Seahorse Extracellular Flux (XFe-96) analyzer (Seahorse Bioscience, Billerica, MA) The XFe-96 measures the concentration of oxygen and free protons in the medium above a monolayer of cells in real time in 96-well formats. Protein concentration was determined for each well using a standard bicinchoninic acid (BCA) based protein assay (Pierce Biotechnology, Rockford, IL, USA). OCR and ECAR values were normalized to μg/protein.

### 4.7. Mitochondrial Function Test

The XF Cell Mito Stress Test quantifies key parameters of mitochondrial function by directly measuring the cell OCR. The XF Cell Mito Stress Test targets components of the electron transport chain (ETC) in the mitochondria by using respiration modulators to reveal these parameters of metabolic function. Three sequential injections of oligomycin, Carbonyl cyanide-4 (trifluoromethoxy) phenylhydrazone (FCCP), plus a mix of rotenone and antimycin A inhibit specific ETC complexes to provide isolated measurements of basal respiration, ATP production, maximal respiration, and non-mitochondrial respiration, respectively. Using these parameters, proton leak and spare respiratory capacity can then be calculated. A specific component of the ETC is targeted by each modulator. Oligomycin can inhibit ATP synthase from complex V. The decrease in OCR following the injection of oligomycin correlates to the mitochondrial respiration, which is associated with cellular ATP production. FCCP is an uncoupling agent that collapses the proton gradient, disrupting the mitochondrial membrane potential. Consequently, electron flow through the ETC is uninhibited, and oxygen is maximally consumed by complex IV. The FCCP-stimulated OCR can then be used to assess spare respiratory capacity, which is a measure of the ability of the cell to respond to an increased energy demand and is defined as the difference between maximal respiration and basal respiration. The third injection is a mix of rotenone, a complex I inhibitor, and antimycin A, a complex III inhibitor. This combination stops mitochondrial respiration, enabling the calculation of non-mitochondrial respiration driven by processes outside the mitochondria.

### 4.8. Glycolysis Stress Test

Glycolysis and glycolytic capacity were determined using the Seahorse Extracellular Flux (XFe-96) analyzer. After 24 h of thiamine incubation, cells were cultured for 2 h without glucose. Three sequential injections of D-glucose (2 g/L), oligomycin (1 μM), and 2-Deoxyglucose (100 mM) provided extracellular acidification (ECAR) that is associated with glycolysis, the maximum glycolytic capacity, and non-glycolytic ECAR, respectively. Glycolysis was defined as ECAR following D-glucose addition; maximum glycolytic capacity was defined as ECAR following oligomycin addition. ECAR following treatment with 2-Deoxyglucose is associated with non-glycolytic activity.

### 4.9. Statistical Analyses

Statistical analyses were performed using SAS for Windows (SAS version 9.4, Cary, NC, USA). Differences between thiamine-treated and vehicle control groups were assessed using linear regression model among groups. A *p*-value of less than 0.05 was considered to be statistically significant. Data are represented as the mean from at least three independent experiments; error bars represent the standard error of the mean (SE).

## 5. Conclusions

The treatment of breast cancer cells with additional high doses of thiamine for 24 h significantly reduces cell proliferation, but has no significant effect on the induction of apoptosis or growth arrest in the cells. The reduction of growth due to thiamine correlates with a reduction in glycolysis and an increase in mitochondrial respiration. These changes correlate roughly with a small thiamine-dependent increase in PDH activity in breast cancer cells.

## 6. Limitations

While our study utilized multiple approaches to examine the effect of thiamine on tumor growth, the study is limited in the type of cell lines used (i.e., breast epithelial cells) and by our single formulation of thiamine (i.e., thiamine hydrochloride). We also recognize that our cell culture system used supra-physiological thiamine concentrations. The thiamine concentration in human plasma has been reported to be in the range of 6.6 nM to 43 nM [23], which is far below the range used in these experiments. In reality, there is no a priori reason to assume that either of our cultured cell models would require physiological thiamine levels or even the same thiamine level since they are different lines, and were not grown in a physiological setting. Each cell line was grown in the medium recommended by the vendor, ATCC, so we have made the assumption that the medium composition for each line is tuned to optimize its growth, and that identical thiamine additions would provide a relevant comparison. Despite this limitation, it was clear that the MCF10A line responded poorly, if at all, to the final thiamine levels, ranging from the baseline media concentration of 0.337 µg/mL (1 µM) to 2.337 µg/mL (7 µM) at the highest addition. In contrast, MCF7 cells responded robustly when thiamine levels were raised from the baseline media value of 0.9 µg/mL (~2.7 µM) to 2.9 µg/mL (8.7 µM) after the highest addition. Future studies may test our hypotheses using a variety of cancer cell lines, more comparison cell lines such as HMECs, and varying thiamine formulations.

Further, while reducing proliferation is important for resolving cancer, it is not sufficient. It is notable that thiamine did not show any significant induction of apoptosis. Therefore, a high dose of thiamine may need to be combined with apoptotic anti-cancer drugs to maximize its ability to inhibit tumor growth.
